# Understanding adherence-related beliefs about medicine amongst patients of South Asian origin with diabetes and cardiovascular disease patients: a qualitative synthesis

**DOI:** 10.1186/s12902-016-0103-0

**Published:** 2016-05-26

**Authors:** Kanta Kumar, Sheila Greenfield, Karim Raza, Paramjit Gill, Rebecca Stack

**Affiliations:** Primary Care Clinical Sciences, University of Birmingham, Birmingham, B15 2TT UK; Faculty of Medical and Human Sciences, University of Manchester, Manchester, M13 9PL UK; Centre for Translational Inflammation Research, Institute of Inflammation and Ageing, University of Birmingham, Birmingham, B15 2TT UK; Rheumatology Department, Sandwell and West Birmingham Hospitals NHS Trust, Birmingham, B18 7QH UK; Centre for Translational Inflammation Research, The School of Immunity and Infection, University of Birmingham, Birmingham, B15 2TT UK

**Keywords:** Adherence, South Asian, Ethnicity, Meta-synthesis, Diabetes, Cardiovascular disease

## Abstract

**Background:**

Prevalence of diabetes and cardiovascular (CVD) disease amongst UK South Asians is higher than in the general population. Non-adherence to medicines may lead to poor clinical outcomes for South Asian patients with diabetes and CVD. To understand the decision making processes associated with taking medicines, a qualitative systematic meta-synthesis exploring medicine taking behaviours, and beliefs was undertaken.

**Methods:**

Four databases (Medline, Embase, Science Citation Index and CINAHL) were searched to identify qualitative studies of South Asian patients taking diabetic medicines. Data were thematic coded and synthesised.

**Results:**

The following themes were identified: [1] beliefs about the need for and efficacy of medicines; [2] toxicity of medicines and polypharmacy; [3] the necessity of traditional remedies versus “western medicines”; [4] stigma and social support; and [5] communication.

**Conclusions:**

South Asians described cultural social stigma associated with diabetes and reported fears about drug toxicity as barriers to taking medicines. Cultural beliefs about traditional remedies and interactions with healthcare professionals also appeared to play a role in the way people made decisions about medicines. Advice should be tailored provided to South Asian patients highlighting the long term consequences of diabetes and CVD.

## Background

The prevalence of diabetes in the UK is estimated to rise to 5 million by 2025 [1]. Type 2 diabetes (T2D) is a major risk factor for cardiovascular disease (CVD) associated morbidity and mortality [2, 3]. The International Diabetes Federation estimated that, in 2003, India had the largest number of people with diabetes in the world (35.5 million) [4], whereas Pakistan had the seventh largest number (6.2 million). Furthermore, people of South Asian origin may have worse prognosis associated with their diabetes when they migrate to western countries [5]. In the UK, South Asians are up to six times more likely than White Europeans to develop diabetes, are more likely to be affected with diabetes at a younger age, and are at greater risk of developing cardiovascular complications [6].

Poor patient adherence to medicines (i.e. not taking medicines as prescribed) [7] can lead to poor clinical outcomes for patients with diabetes and CVD [8–10]. Studies have found that patients’ beliefs about medicines and their perception of their illness contribute towards poor adherence [11, 12]. However, little is known how cultural beliefs shape the way that medicines prescribed for the treatment of diabetes are taken by people of South Asian origin, either in South Asian countries or in countries to which they have migrated.

This paper synthesises the qualitative literature which has explored behaviour towards medicines and beliefs about medicines amongst South Asians (i.e. those from India, Pakistan or Bangladesh) with diabetes and CVD.

## Method

**Fig. 1:**
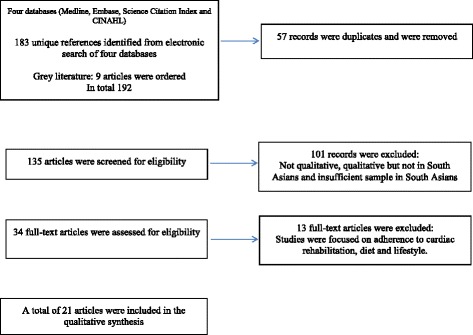
Flow of studies through review. Inclusion criteria: • Papers were restricted to the English language. • Qualitative studies using an interpretative method to analyse and report data on adherence to medicines in South Asian adults (>18 years). • Diagnosed with diabetes or CVD. Exclusion criteria: • Papers excluded if they did not specify which data were derived from people of South Asian origin.• Editorials, conferences proceedings, letters, news articles, government reports and practice guidelines were reviewed and were excluded if they did not have primary data

Standard systematic review guidelines were used [13]. Four databases (Medline, Embase, Science Citation Index and CINAHL) were searched from January 1980 to June 2015 (Fig. 1) using a combination of search terms (Table 1). Google Scholar and Index Medicus for the Southeast Asian Region database were searched to identify research conducted in South Asian countries. The systematic review search structure was verified by a senior librarian based at the Sandwell and West Birmingham Hospitals NHS Trust library.

**Table 1:** An example of order construct

Study	Theme/category	Data extract (first order construct)	Data extract (second order construct)	Conclusion (synthesis)
Lawton et al., 2005 [21]	Adjusting of drugs/symptoms severity	“....And now in the morning I take three pills, sometimes two, meaning I check it (blood glucose) and according to that I do or don’t take all the pills”.	Popular ideas about medicines, derived from the Indian subcontinent, may have informed the ways respondents perceive and take their oral hypoglycaemic agents (OHAs). On the Indian subcontinent, people commonly self-medicate, make selective use of prescribed drugs, and abandon drugs that do not provide prompt relief of symptoms. This might explain why some respondents adjusted their OHAs without seeking medical approval and according to the presence or absence of symptoms.	Symptom control significantly influenced adherence to treatment. Patients’ health beliefs and medication adherence may be affected by the severity of disease. Patients make critical decisions that affect the therapeutic outcome of their disease. Patients’ beliefs about disease and medications may be crucial to their intentional adherence behaviours and may be very different to those of the health professional.
Lawton et al., 2006 [22]	God/fatalistic view	“....God has given me these diseases, and they will never go away, you just get more and you cannot do much about it”.	Whilst fatalism has been attributed to belief in God’s will, which is a strong feature of the Muslim, Hindu and Sikh religions our findings suggest that call/recall systems may reinforce patients’ perceptions that they are not responsible for disease self-management, when they have migrated from a country where they are expected to seek out medical care.	Generally south Asian patients were more likely than their European counterparts to contextualise their diseases in relation to their religion beliefs. This often resulted in lack of motivation to improve their disease.

Inclusion and exclusion criteria were applied as follows; *Inclusion criteria:* papers were restricted to the English language only as no funding was available to support the translation of non-English language papers. Qualitative studies using an interpretative method to analyse and report data on adherence to medicines in South Asian adults (>18 years), diagnosed with diabetes or CVD were included. *Exclusion criteria:* papers were excluded if they did not specify which data were derived from people of South Asian origin. Editorials, conferences proceedings, letters, news articles, government reports and practice guidelines were reviewed and were excluded if they did not have primary data (e.g. patient quotes).

### Search terms

Search terms used for identify the studies included; Diabetes mellitus diabetes; OR glycaemia; OR insulin; OR oral hypoglycaem; OR blood glucose; OR hypoglycaemic agents; OR hyperglycaemia. Cardiovascular diseases; OR hypertension; OR hyperlipidemia; OR oral cholesterol; OR blood pressure; OR heart disease; OR heart failure. India south Asia Pakistan Bangladesh Culture. Cultural and religious beliefs, values, and customs, can influence illness perceptions and medication adherence therefore qualitative studies were searched including the following; adherence; OR adherent; OR compliance; OR concordance; OR persistent; OR retention; OR drop out; OR medication; OR attitude to health; OR health beliefs.

### Data extraction

The Critical Appraisal Skills (CASP) Programme quality criteria were used for data extraction [14]. Two reviewers (KK and RS) independently extracted data. A meta-synthesis approach was used to synthesise data [15] which involved the translation of findings into first, second and third order themes across the different studies. Table 1 shows an example of first, second and third order synthesis. To identify first order themes, quotations where interviewees expressed their views about adherence to medicines were extracted. To identify second order themes, sections of the results of the authors’ interpretation of findings were extracted. The third order of themes were developed through combining first and second order themes [15]. Articles were analysed using an approach informed by thematic analysis and grounded theory framework [16, 17] to systematically identify shared concepts and themes [15]. KK and RS then conducted a second review of the papers and identified whether they contained text about barriers to medication adherence. At each stage of data abstraction, the reviewers discussed the studies to achieve consensus regarding the identification and coding of themes. Any discrepancies were discussed between the researchers and resolved.

## Results

Twenty-one publications were included in the final analysis (Table 2). Sixteen studies were UK based [18–32], three from Canada [33–35], and one from Norway [36]. Only one study [37] was based in the South Asian region. Seventeen studies focused on diabetes, three on CVD and one was on both diabetes and CVD. The majority of the patients included in the studies were female. The following themes were identified: [1] beliefs about the need for and efficacy of medicines; [2] toxicity of medicines and polypharmacy; [3] stigma and social support; [4] the necessity of traditional remedies versus western medicines; and [5] communication. These themes are described in detail below and are illustrated with quotations extracted from the original papers.

**Table 2:** Study characteristics

References	Aim of the paper	Country	Sample characteristics	Quality method and analysis used	Quality check
			[1] Number		
			[2] Disease		
			[3] M/F		
Bissell et al., 2004 [18]	To explore barriers to accomplish a re-framed model of health care in English- speaking patients of Pakistani origin with type 2 diabetes.	UK	[1] Twenty one patients	In-depth semi-structured interviews, analysis using grounded theory.	Transcripts were independently reviewed by research team.
			[2] Patients diagnosed with diabetes		
			[3] Gender not reported		
Fagerli et al., 2005 [36]	To explore how ethnic minority persons with diabetes experience dietary advice given by Norwegian health workers.	Norway	[1] Fifteen patients	In-depth semi-structured interviews, analysis using grounded theory.	Transcripts were independently verified by interpreters.
			[2] Patients diagnosed with diabetes		
			[3] M = 4/F = 11		
Fleming et al., 2008 [38]	To explore the influence of culture on type 2 diabetes self-management in Gujarati Muslim men.	Canada	[1] Five patients	Case study approach. Analysis using theory building and fieldwork.	None reported.
			[2] Patients diagnosed with diabetes		
			[3] M = 5		
Galdas et al., 2010 [33]	To explore the cardiac rehabilitation experiences of Punjabi Sikh patients post myocardial infarction.	Canada	[1] Fifteen patients	In-depth semi-structured interviews, analysis using grounded theory.	Transcripts were independently reviewed by research team.
			[2] Patients diagnosed with CVD		
			[3] M = 10/F = 5		
Keval et al., 2009 [19]	To explore South Asian Hindu Guajarati speaking people’s experience of type 2 diabetes.	UK	[1] Eighteen patients	In-depth semi-structured interviews, analysis using grounded theory.	Transcripts were independently reviewed by research team.
			[2] Patients diagnosed with diabetes		
			[3] M = 10/F = 8		
King et al., 2006 [34]	To explore the influence of gender on managing coronary artery disease.	Canada	[1] Eighteen patients	In-depth semi-structured interviews, analysis using grounded theory.	Transcripts were independently verified by interpreters.
			[2] Patients diagnosed with CVD		
			[3] M = 10/F = 8		
Lawton et al., 2005 [20]	To explore British Pakistani and British Indian patients’ perceptions and experiences of taking oral hypoglycaemic agents.	UK	[1] Thirty two patients (fifteen South Asian)	In-depth semi-structured interviews, analysis using grounded theory.	Transcripts were independently reviewed by authors.
			[2] Patients diagnosed with diabetes		
			[3] M = 4/F = 11		
Lawton et al., 2005 [21]	To explore Pakistani and Indian patients’ experiences of, and views about, diabetes services.	UK	[1] Thirty two patients	In-depth semi-structured interviews, analysis using grounded theory.	Transcripts were independently verified by research team in addition software for qualitative data analysis was used.
			[2] Patients diagnosed with diabetes		
			[3] M = 15/F = 17		
Lawton et al., 2006 [22]	To explore patients’ perceptions and experiences of under-taking physical activity amongst people of Pakistani and Indian origin with type 2 diabetes.	UK	[1] Thirty two patients	In-depth semi-structured interviews, analysis using grounded theory.	Transcripts were independently verified by research team and interpreters.
			[2] Patients diagnosed with diabetes		
			[3] M = 17/F = 15		
Lawton et al., 2007 [23]	To explore patients’ perceptions and understanding of disease causation.	UK	[1] Thirty two patients (fifteen South Asian)	In-depth semi-structured interviews, analysis using grounded theory.	Transcripts independently were verified by authors.
			[2] Patients diagnosed with diabetes		
			[3] M = 4/F = 11		
Lewis 2007 [24]	To examine the lived experience and cultural illness explanations of a sample of British Indian diabetic patients living with leg and foot ulcers.	UK	[1] Sixteen patients	In-depth semi-structured interviews, analysis using Greens’ Framework used to code data. (framework not explained).	None reported.
			[2] Patients diagnosed with diabetes		
			[3] Gender not reported		
Meetoo 2004 [25]	To examine the dietary pattern of self-care for a group of Asian and Caucasian diabetes patients.	UK	[1] Twenty five patients	In-depth semi-structured interviews, analysis using grounded theory.	Transcripts were independently reviewed by research team.
			[2] Patients diagnosed with diabetes		
			[3] M = 8/F = 17		
Meetoo 2005 [26]	To explore the Explanatory models of diabetes by a group of Asian and Caucasian patients.	UK	[1] Twenty five patients	In-depth semi-structured interviews, analysis using grounded theory.	Transcripts were independently reviewed by research team.
			[2] Patients diagnosed with diabetes		
			[3] M = 8/F = 17		
Oliffe et al., 2010 [35]	To describe the connections between masculinities and diet among senior Punjabi Sikh Canadian immigrant men.	Canada	[1] Eighteen patients	In-depth semi-structured interviews, analysis using grounded theory.	Transcripts were verified by interpreters and NVivo software was used.
			[2] Patients diagnosed with CVD		
			[3] M = 18		
Rafique et al., 2006 [37]	To assess the needs, awareness and barriers to diabetes education or self-management in diabetic patients.	Pakistan	[1] Twenty seven patients	In-depth semi-structured interviews, analysis using grounded theory.	None reported.
			[2] Patients diagnosed with diabetes		
			[3] M = 11/F = 16		
Rhodes et al., 2003 [27]	To examine the experience of diabetes and local services in Bangladeshi diabetic patients.	Canada	[1] Eighteen patients	In-depth semi-structured interviews, analysis using grounded theory.	Transcripts were independently verified between authors. Member checking was undertaken with authors checking codes.
			[2] Patients diagnosed with diabetes		
			[3] Gender not reported		
Rhodes et al., 2003 [28]	To examine access from the point of view of Bangladeshi diabetic patients.	UK	[1] Twelve patients	In-depth semi-structured interviews, analysis using text detective software package.	None reported.
			[2] Patients diagnosed with diabetes		
Singh et al., 2012 [29]	To explore experiences of UK based South Asian and White patients with diabetes.	UK	[1] Twelve patients	In-depth semi-structured interviews, analysis using phenomenology	Transcripts were independently verified by authors.
			[2] Patients diagnosed with diabetes		
			[3] M = 6/F = 6		
Stack et al., 2008 [30]	To explore perceptions towards multiple medicines expressed by people managing co-morbidities.	UK	[1] Three patients	In-depth semi-structured interviews, analysis using grounded theory.	Transcripts were independently verified by authors.
			[2] Patients diagnosed with diabetes & CVD		
			[3] Gender not reported		
Stone et al., 2005 [31]	To explore the experience and attitudes of primary care patients with diabetes living in UK.	UK	[1] Fifteen patients	In depth semi-structured interviews, analysis using grounded theory.	Transcripts were independently verified between two researchers.
			[2] Patients diagnosed with diabetes		
			[3] M = 6/F = 9		
Wilkinson et al., 2012 [32]	To explore renal complications of diabetes from the patients’ perspective.	UK	[1] Twenty five patients	In-depth semi-structured interviews, analysis using grounded theory.	Transcripts were independently verified between authors. NVivo software used to code data.
			[2] Patients diagnosed with diabetes		
			[3] M = 16/F = 9		

For studies where patients from multiple ethnicities were included the sample characteristics column only includes data relevant to the South Asian patients studied. Where it was not possible to identify which patients were of South Asian origin such papers were excluded from the review

### Beliefs about the need for and efficacy of medicines

Many patients of South Asian origin regarded medicines for the treatment of diabetes and CVD as necessary. The danger of not taking medicines was recognised and the roles of medicines in reducing the risks associated with illness were described (Quotes 1 and 2). Patients also recognised that it was necessary to take these medicines on a long term basis (Quote 3).

**Table:** 

[Quote 1]*“If I didn’t take them then I would be in danger.”* Male of Pakistani origin diagnosed with diabetes: UK study [20].
[Quote 2]*“Once you start on these then you have to be on them for the rest of your life. So either you do that, or you risk dying. So you have no choice but to take the medicine.”* Female of Pakistani origin diagnosed with diabetes: UK study [20].
[Quote 3]*“My health depends on these and I am continuing to take my medications now.”* Female of Indian origin diagnosed with CVD: Canada Study [33].

Not only were medicines seen as necessary but they were also viewed as effective. However, the perceived effectiveness of medicines varied depending upon where they had been prescribed. Patients who had migrated to the UK described the medicines they received in the UK as being more effective than those they would have received in places like India and Pakistan (Quotes 4 and 5). These pro-western medicine thoughts were seen also in those who also tried alternative medicines. Patients appeared to place a high value on evidence based medicines prescribed in the West (Quote 5). However, traditional and alternative medicines that originated from the South Asian region were also valued, and held by some in the same regard as medicines prescribed in the west (Quote 6).

**Table:** 

[Quote 4]*“I don’t think you can get the same kinds of medicine that you can get here, you know, like metformin. This is one of the most important drugs to take for it.”* Female of Indian origin diagnosed with diabetes: UK study [20].
[Quote 5]*“See, in Pakistan, the medications are not right, they’re just a waste of time, waste of money. I mean these [referring to hypoglycaemic agents] are the real stuff. These are what really work.”* Male of Pakistani origin diagnosed with diabetes: UK study [20].
[Quote 6]*“At the moment I’m eating ‘Methi’ [fenugreek] because I believe in Ayurvedic medicine. But I’m also very pro-western medicine as well – it’s a question of trial and error.”* (gender not stated) South Asian of Indian origin diagnosed with diabetes: UK study [19].

However, while some spoke of the need for their medicines, many patients reported having missed doses of their medicines intentionally (Quotes 7 and 8). In some cases, somatic cues such as “feeling fine” were given as reasons for not taking medicines (Quotes 7 and 9). Some felt that there was less benefit in taking lipid-lowering medicines as they did not notice any symptomatic difference whether they took the medication or not (Quote 10).

**Table:** 

[Quote 7] *“Sometimes you do say that to yourself, you know, you say to yourself, ‘Oh I feel fine and I’ll take one today, I won’t take two.”* Female of Pakistani origin diagnosed with diabetes: UK study [20].
[Quote 8]“I don’t take my tablets on many times [laughs].” Male of Pakistani origin diagnosed with diabetes: UK Study [20].
[Quote 9]*“They said that I need to take three, but for the last three months I’m just taking them twice a day. It’s just when I feel I’m tired I take another one. If I’m fine then I won’t.”* Male of Pakistani origin diagnosed with diabetes: UK study [20].

Another factor that influenced decisions not to take medicines was perceived symptom severity (Quote 10). Some adjusted their diabetic medicine, according to what they ate (Quote 11).

**Table:** 

[Quote 10] *“I don’t find any difference but because they say that my cholesterol level is slightly higher that it should have been – I think if I don’t take I, I don’t find any difference”* Male of South Asian origin diagnosed with CVD: UK Study [30].
[Quote 11]*“Sometimes I will take two when I don’t spread too much jam on my toast or even sometimes I don’t even spread any. If I feel like a bit of a pleasure then I will put some on and then I will take the extra tablets.”* Male of Pakistani origin diagnosed with diabetes: UK study [20].

Some patients made a conscious decision to stop their treatment during social gatherings (Quote 12). South Asian patients often stopped their medicines to fully partake in activities such as weddings (Quote13).

**Table:** 

[Quote 12]*“I never comply with my treatments at weddings and parties....”* Female of Indian origin diagnosed with diabetes: UK study [25].
[Quote13] *“I visit families and friends a lot of the time. I don’t comply especially when I go to weddings and when I visit friends and relatives. At other time I don’t always comply with treatment within my own house.”* Female of Indian origin diagnosed with diabetes: UK study [26].

In some cases, patients were influenced by other family members’ experiences of taking medicines. Some patients felt that the complications of their illness were unavoidable and inevitable even if medicines were taken (Quote 14). A few patients used self-management strategies and life style changes as a rationale for stopping their medicine (Quote 15).

**Table:** 

[Quote 14]*“My father also had diabetes. He used to be very adherent [to diet and medication]. But despite this he died of a stroke. Since then, I have become non-adherent. I think, what is the point?.”* Male of Pakistani origin diagnosed with diabetes: UK study [37].
[Quote 15]*“Last time I went to Pakistan, I went for ten months and I brought with me my blood-sugar monitor and two kinds of tablets. The first five months I used the medications, but not the last five. That’s because I went hunting every second day and walked for 15 to 20 km. In the evenings when I checked, my blood sugar was ok, and I didn’t have to use the tablets.”* (Gender not stated) patient of Pakistani origin diagnosed with diabetes: Norway study [36].

### Toxicity of medicines and polypharmacy

Some patients were concerned about increasing numbers of prescribed medicines being added to their treatment regimens, adding to their fears of toxicity (Quote 16). Concerns were expressed about potential side effects of medicines, and beliefs about toxicity (with medicines viewed as poisons) (Quote 17).

**Table:** 

[Quote 16]*“Initially it was just two metformins a day, and then it was increased to four by the doctor. And then there’s blood pressure tablets to take and then aspirins and so on. So it all adds up and, you know, if you take seven, eight pills a day and you wonder [laugh] is it the right thing? This can’t be good for me in the long run these can be poisons.”* Male of Indian origin diagnosed with diabetes: UK study [20].
[Quote 17]*“… like the thought of something going wrong with my kidneys, like something going wrong with your lungs or your heart, it’s scary. So I think I would have - I probably wouldn’t even be on insulin now.”* (Gender not stated) South Asian of Indian origin diagnosed with diabetes: UK Study [32].

A number of patients feared that taking too many medicines, would lead to death (Quote 18). South Asian patients suggested that taking too many medicines caused them to feel “dull” and “dry” (Quotes 19 and 20). In instances where toxicity had been experienced, anger was expressed. Patients felt frustrated when one tablet resulted in a complication to control which they had to take an additional medicine (Quote 21).

**Table:** 

[Quote 18]*“Yes, they told me to take it every day, but I said ‘do I want to die by taking it every day…I don’t want to die by taking so many.”* Female of Pakistani origin diagnosed with diabetes: UK study [20].
[Quote 19]*“Already I am dull, my body is, by taking so many tablets.”* Female of Pakistani origin diagnosed with diabetes: UK study [20].
[Quote 20]“*Sometimes I don’t take them, you know, they make you dry if you take too many.”* Female of Pakistani origin diagnosed with diabetes: UK Study [20].
[Quote 21]*“For my asthma, they gave me tablets and they were sweet tablets [steroids], and I had to take eight tablets all at once…I stayed [in hospital] for a week and they gave me all those tablets, and because of that I got sugar…I was angry that I got sugar because of their medication.”* Female of Indian origin diagnosed with diabetes: UK study [23].

### Stigma and social support

Stigma and social support had a major influence on medicine taking. Patients were reluctant to disclose their use of insulin to their families and community. Some patients were told by family members not to tell anyone that they were taking insulin, which made it difficult to take the medication during social occasions. In some cases, coping strategies were developed to ensure that the insulin was not used during social situations, including taking insulin before attending the social function (Quote 22). The social stigma attached to diabetes and insulin therapy was associated with embarrassment on the part of the patient. This made some patients reluctant to initiate insulin therapy (Quote 23). Patients felt that illnesses such as diabetes were not socially acceptable within South Asian communities (Quote 24).

**Table:** 

[Quote 22]*“When I am back in Pakistan they (family) don’t let me tell anybody that I have it, which makes it very difficult for me when I go out. If I am going around somebody’s house for a meal, they make me do the injection before I go. I can sit there and they won’t have their meal ready till two hours later and I will just have to keep popping myself with coke…”* (gender not stated) Patient of Pakistani origin diagnosed with diabetes: UK study [29].
[Quote 23]*“I got a shock when they put me on insulin … I asked doctors to give me two weeks to decide whether I want to start taking insulin or not, it is not difficult in the personal sense … it is more because of our culture and community. People look at you and go, ‘Oh God! Is he taking insulin?’ … people feel that you have a very dangerous kind of disease …it is really embarrassing.”* (gender not stated) South Asian of Indian origin diagnosed with diabetes: UK study [29].
[Quote 24]*“In our culture you’re not wanting to know that you‘ve got any kind of disease like diabetes which is why we don’t want the injections.”* Male of Pakistani origin diagnosed with diabetes: UK study [22].

For people from a South Asian background, diabetes and insulin were viewed in very negative terms, and it was expressed that they were not culturally accepted. In contrast, in some situations close family were felt to be positive agents in supporting self-management and often helped to facilitate medicine taking (Quote 26). Some patients had a family support mechanism which they felt helped them to take medicines (Quote 27).

**Table:** 

[Quote 26]*“I feel there is no life without wife. After a certain age there is a desperate need for a partner… they will remind you and say, ‘have you taken your insulin?’ or ‘take your insulin and in the mean time I will prepare food for you and lay it on the table’… this way, together you can look after diabetes better.”* (gender not stated) Patient of Pakistani origin diagnosed with diabetes: UK study [29].
[Quote 27]*“My daughter is a nurse. I learned a lot from my daughter. At first, she used to do all the monitoring and injecting and things, but now I can do them myself.”* Male of Pakistani origin diagnosed with diabetes: Pakistan study [37].

### The necessity of traditional remedies versus Western medicines

This theme covers a different concept of medicine taking to the theme “beliefs about the need for and efficacy of medicines” in that patients experimented with traditional remedies in parallel with Western medicines. Many patients made use of traditional remedies alongside their Western medicines and did not associate “herbal” medicines with the perceived toxicity associated with medicines prescribed by the doctor (Quotes 28–32). Not only were traditional remedies viewed as necessary, they were also viewed as effective, with one patient describing how traditional remedies were efficacious in controlling cholesterol levels (Quote 28). Patients also described a number of positive attributes of traditional remedies, including the fact that these remedies “make quite a difference” (Quote 29), have no side effects (Quote 30), provide balance (Quote 30) and are natural (Quote 31). In some cases patients described traditional remedies as being better at tackling illnesses (Quote 31) and able to control the adverse consequences of illness (Quote 32).

**Table:** 

[Quote 28]*“There are some things like ginger and garlic that we use. These two things are good for a man’s health. The more you use it the better it is. They reduce cholesterol, it makes a difference to heart attack too.”* Male Immigrant of Indian origin diagnosed with diabetes: Canada study [35].
[Quote 29]*“I always check my levels, and then take my tablets, and if I need to I’ll have some chocolate or something. I’ll also take some ‘kurvat ni phaki’ [bitter powder] … this makes quite a difference to me. This is from Dubai … whatever happens I always take my medication … I use ‘karela’* [bitter gourd] *as well.”* Female of Indian origin diagnosed with diabetes: UK Study [19].
[Quote 30]*“It’s fine, there’s no side-effects, it’s all herbal … there should be more information about these things. We also use Neem [Azadirachta Indica] powder, which we used to use for malaria in India, we got this from here. We alternate these remedies, to balance the different things.”* Male of Indian origin diagnosed with diabetes: UK Study [19].
[Quote 31]*“It’s green medicine. It’s natural medicines and it has a reputation for maybe tackling conditions that western medicines are not so used to.”* (gender not stated) Patient of Indian origin diagnosed with diabetes: UK Study [24].
[Quote 32]*“First I take a quarter spoon of turmeric in warm water, then I use Meth [fenugreek], and then I take ammo [again], which reduces the amount of gas we get … turmeric is an antibiotic, oftentimes diabetics get this and that, and I still don’t have any infections from where I have been hurt.”* Female of Indian origin diagnosed with diabetes: UK study [19].

South Asian patients were open about the fact that traditional and herbal remedies were widely available. Media within the South Asian community often portrayed these remedies in a positive light (Quote 33). Family and friends were important in decisions to use alternative medicines, and in some cases would supply these medicines (Quote 34).

**Table:** 

[Quote 33]*“There was an article … in a newspaper which we get from India, and my niece sent the Jambu [rose apple] powder from India…but you can get this information from Gujarati newspapers here as well. ‘Karela’* [bitter gourd] *we also use, both as a curry and tablets.”* Male of Indian origin diagnosed with diabetes: UK study [19].
[Quote 34]*“Well, we’re from India, so my mother and others used them. My brother was always using these medicines … he used to write to me with advice and send them to me.”* Female of Indian origin diagnosed with diabetes: UK study [19].

Some patients placed more faith in Western medicines than traditional remedies (Quotes 35 and 36). One patient spoke of how they had moved away from traditional remedies to accept medicines prescribed by the doctor (Quote 35), while others spoke of how traditional remedies played a large role in the management of diabetes in South Asian countries (Quote 36).

**Table:** 

[Quote 35]*“I used to take juice of bitter gourd for sugar [diabetes] problems….Now I have stopped that. I have been given a tablet by the doctor and eat that once a day.”* Male Immigrant of Indian origin diagnosed with diabetes: Canada study [19].
[Quote 36]*“Homeopathy in India is very big and homeopaths are everywhere. A lot of people go to them and don’t go to a doctor. My wife said to me you should go to a homeopath but actually I don’t believe in that,. I believe in medicines.”* (gender not stated) South Asian of Indian origin diagnosed with diabetes: UK study [19].

### Communication

Health professionals’ communication styles influenced the way patients viewed the disease process and medicines (Quotes 37 and 45). Some patients felt that they were not always fully informed about disease management and how the medicines would help to control their symptoms (Quote 39). Some expressed a lack of engagement with the decisions that many doctors made and did not understand the treatment plan (Quotes 39–42).

**Table:** 

[Quote 37] *Daughter: “They have done it only recently. My mother had an appointment in September, but I could not go with her because I had personal problems from the surgery, they never bothered to chase that appointment from September to January. I myself took my mother to the surgery, that when they found my mother HbA1c reading was 12.9. The tablets my mother was taking, she was supposed to take one tablet daily, but she was taking the same tablet twice, which she should not have done.”* South Asian of Indian origin diagnosed with diabetes: UK study [27].
[Quote 38]*“One thing is, if you were having a side effect from your medicine, you could discuss it with your doctor, or the nurse. Yes, yes, I see. But this has happened and I talk with doctor about it. And he tell me it will pass and it did. PB. So you can already do that? Oh yes, if want to ask questions, then I do already. I can do that with doctor. He say we can do that.”* (gender not stated) South Asian of Indian origin diagnosed with diabetes: UK study [18].
[Quote 39]*“I’ve seen it happen. They’ll be waiting to ask questions about their medicines or what have you and then not feel like they can when they get in there. I’ve felt like that myself, haven’t you? It’s like you don’t think you can ask any questions when you get in the room with the doctor.”*(gender not stated) South Asian of Indian origin diagnosed with diabetes: UK study [18].
[Quote 40]*“The doctor had then taken lots of tests and he gave the medicines. He didn’t say anything in particular about how to take care of my heart he just gave the medicines.”* (gender not stated) Sikh Immigrant of Indian origin diagnosed with CVD: Canada study [34].
[Quote 41]*“We take tablets, but how are we supposed to know if it’s in control or not? I’ve got this stick thing to measure it with and I have also got this machine and with that you know what it is, whether it is 7.5 or 8.5 or whatever.”* Male of Pakistani origin diagnosed with diabetes: UK Study [31].
[Quote 42]*“He was giving me medication but wasn’t asking me for any urine sample or anything.”* Female of Pakistani origin diagnosed with diabetes: UK study [28].

Some patients drew a comparison between receiving diabetic care in countries such as the UK and South Asian countries (Pakistan and India) where paying for medicines was suggested as a barrier to adherence to the medication regimen (Quote 43). It was suggested that patients with better financial resources were able to receive better treatment in Pakistan (Quote 44). Patients often compared and contrasted different health systems: for example, patients felt that the healthcare system in the UK was more trustworthy than countries such as India and Pakistan. This view had an impact on the way patients communicated with UK doctors about their medicines, and followed advice (Quote 45).

**Table:** 

[Quote 43]*“It depends how rich you are, how much money you’ve got for the medication......they would go to the doctor but paying for the medication or being told you will have this and you’ve got to pay this much every month for a tablet, it’s highly unlikely that they’re going to stick to that regimen.”* Male of Pakistani origin diagnosed with diabetes: UK study [38].
[Quote 44]*“The poor over there (Pakistan) die, because they can get no treatment. Doctors’ pockets over there are so big, they’re full of notes.”* Female of Pakistani origin diagnosed with diabetes: UK study [21].
[Quote 45]*“You know how it is there, our doctors don’t really pay attention. They are more concerned with the amount of money they are making. First they will give you a lighter medication, which will make you go back to them again and again until they give you something else. And by that time, you will be feeling better anyway.”* Male of Pakistani origin diagnosed with diabetes: UK Study [20].

## Discussion

This systematic meta-synthesis review has identified five interacting themes related to adherence to medicines for diabetes and CVD described by people of South Asian origin. Concerns about medication toxicity were described as a reason for not taking medicines as prescribed, though beliefs about the need for and efficacy of medicines were identified as important. Beliefs in traditional remedies and patient-health professional interaction appeared to play an important role. Patients’ beliefs about traditional remedies caused some to doubt the efficacy and necessity of prescribed medicines.

The beliefs about medicines of diabetic and CVD patients identified in this systematic meta-synthesis review were similar to those identified in South Asian patients with other chronic disease for example cancer [39–41],asthma [42], renal failure [43, 44], and rheumatoid arthritis [45, 46]. Furthermore, beliefs about medicines and the use of traditional remedies play an important role in medication adherence. This has been noted more amongst female than male South Asian patients [47]. The use of traditional remedies influences patients’ decisions to take “western” medicines. Furthermore, patients have been shown to delay in seeking medical treatment even after noticing symptoms hoping that traditional remedies will alleviate their symptoms. [39] Moreover, South Asian patients have been shown to feel more satisfied with traditional remedies than with “western” medicines [48].

Our review identified that patients were less concerned about the long-term management of their underlying illness. This may be associated with a lack of knowledge about the long-term consequences of the illness and cultural stigma attached to illness representation, particularly diabetes. Evidence in other disease areas such as cancer has shown that in South Asian patients, coping with chronic illnesses is more centred on family support and spiritual beliefs [49], compared with patients from other ethnic groups [24 African American, 34 Asians, 26 Latinas and 18 “Caucasian” patients] which could have a potential impact on treatment choices. For example, emerging research shows that women whose views are centred on spiritual beliefs tend to cope less well with chronic diseases compared to those whose views are based on understanding the chronic disease [40, 41]. South Asian women’s challenges in coping with chronic illness may differ from the general population and in addition language barriers and societal stigmas may also influence treatment decisions [40].

From our review, it appeared that diabetic patients were often unaware of the impact of fluctuating poor adherence on the long-term consequences of uncontrolled diabetes. Again these concepts were linked with the perceived efficacy of and concerns about medicines. Similar themes have been reported in another review of studies involving patients from countries including the USA, UK, Brazil, Sweden, Canada, Finland, Netherlands, South Korea and Spain [50] who had also expressed views about the need for and efficacy of medicines, intentional non-adherence to medicines, toxicity of medicines and polypharmacy and the necessity of traditional remedies [50]. Furthermore, that review of studies involving patients of European background [50], found that hypertensive patients with high adherence had an understanding of hypertension which mapped onto the biomedical model. However, this concept was not found in our review. One of the explanations for this could be that many South Asian patients did not understand the disease process and were unclear about the reasons for taking their medicines. Patients’ understanding and acceptance of the diagnosis is an important component of medicine taking and disease self-management. Cultural influences and social misconceptions of diseases have been reported to hinder patients’ understanding of disease in South Asian patients [51].

Our findings suggest that making use of theoretical frameworks could be helpful to enable health professionals to fully understand the factors influencing adherence amongst South Asians. This has been shown in other studies [52–54] Theoretical frameworks such as the Self-Regulation Model (SRM) [53] may enable health professionals to place health beliefs into context and develop interventions to change medication behaviour. The SRM [53] is a psychological model that attempts to capture people’s experience during illness by emphasizing five illness cognitions: illness identity, illness cause, illness timeline, illness consequences and illness controllability [53]. Some of the dimensions within the SRM model if used quantitatively may help to tease out causal and control beliefs about illness and treatment. It therefore offers different interpretation of the processes behind adherence behaviour. Furthermore, researchers might be able account for diabetes related beliefs using such measures as the SRM may help identify the key barriers to medication adherence and effective self-management. It is important to note, however, that SRM have not been widely used in South Asian patients in the context of investigating medication adherence but its use in non-South Asian patients has been documented [53]. The use of SRM model has been made in conditions such as HIV [54], inflammatory bowel disease [55] and in patients with hypertension [12] to predict medication behaviour.

### Strengths and weakness

This review synthesised a range of international qualitative studies exploring beliefs about medicine taking among people of South Asian origin with diabetes/CVD. However, we identified only one study conducted in South Asia. Therefore, the findings of this review have been strongly influenced by the perceptions of people who have migrated to western countries. A limitation of this review was that some South Asian journals were not indexed in Medline, Embase or CINAHL. For example, “Cardiology and angiology: an International Journal” was included in Index Medicus but was not indexed by Medline, Embase or CINAHL: therefore, we were not able to identify all potentially relevant research studies published in the South Asian region. Another limitation of the review was that there were few qualitative studies exploring CVD medicines taking in people of South Asian origin field therefore perspectives of South Asian patients with CVD may not be fully captured by our review. Further qualitative research is warranted in this high-risk population.

## Conclusion

This review has identified a range of beliefs which play a key role in influencing adherence to medicines. Theoretically based interventions based on the SRM may benefit South Asian patients so they can develop a rationale for taking their medication.

## Abbreviations

CVD: cardiovascular disease
T2D: type 2 diabetes
SRM: self-regulation model
